# Heme-Binding Groups in Cytochrome P450 Inhibitors: Coordination Mechanisms, Evidence Standards and Implications for CYP19A1 Ligand Design

**DOI:** 10.3390/molecules31203656

**Published:** 2026-10-13

**Authors:** Paweł Kozyra, Maciej Masłyk

**Affiliations:** 1 Department of Biomedical and Analytical Chemistry, The John Paul II Catholic University of Lublin, St. Konstantynów 1j, 20-708 Lublin, Poland; pawel.kozyra@kul.pl; 2 AI Drug Discovery Center, The John Paul II Catholic University of Lublin, St. Konstantynów 1j, 20-708 Lublin, Poland; 3 Department of Molecular Biology, The John Paul II Catholic University of Lublin, ul. Konstantynów 1i, 20-708 Lublin, Poland

**Keywords:** aromatase, aromatase inhibitors, CYP19A1, coordination mechanisms, heme, chelation, heme-binding groups, drug discovery, cytochrome P450

## Abstract

Cytochrome P450 (CYP) enzymes are heme-thiolate monooxygenases whose inhibition can result from direct reversible axial ligation of the distal heme iron, perturbation of the axial-water environment, or metabolism-dependent heme interactions. This focused review critically examines the chemical classes used as putative heme-binding groups in small-molecule CYP inhibitors, with emphasis on imidazoles, 1,2,4-triazoles, pyridines, 1,2,3-triazoles, sulfur-containing heterocycles, hydroxamates, nitriles and isonitriles. We discuss the evidential limits of ligand-induced type II spectra, distinguish direct parent–ligand coordination from mechanism-based inactivation, and outline an evidence hierarchy integrating enzymology, UV–Vis spectroscopy, EPR-based methods, structural biology and QM/MM calculations. CYP19A1 is used as a medicinal-chemistry case study illustrating how donor identity, binding geometry, scaffold recognition and selectivity against drug-metabolising and steroidogenic CYP enzymes must be optimized concurrently. We propose practical design and validation principles for the development of selectively heme-directed CYP19A1 inhibitors.

## 1. Introduction

Cytochrome P450 (CYP) enzymes are heme-thiolate monooxygenases that oxidise endogenous substrates, xenobiotics, and drugs. Their catalytic centre contains protoporphyrin IX iron ligated proximally by a conserved cysteinate thiolate. In the substrate-free ferric state, heme iron is commonly six-coordinate and low-spin, with water at the distal axial site. Binding of many substrates displaces water and shifts the equilibrium towards a five-coordinate, high-spin ferric state, facilitating electron transfer and oxygen activation [1,2,3]. The distal coordination site is an exploitable feature in CYP inhibitor design. Appropriately positioned Lewis-basic groups may interact with heme iron through direct axial coordination or water-mediated arrangements. Imidazole-, triazole-, and pyridine-containing inhibitors commonly stabilise low-spin ferric complexes and produce type II difference spectra. These interactions can inhibit catalysis by altering the heme coordination environment and access to the catalytic cycle; the consequences for electron transfer and reduction kinetics are ligand- and isoform-dependent [4,5,6,7].

However, a type II spectrum does not prove a persistent direct Fe–N bond [4]. Its properties depend on donor electronics, ligand orientation, steric accessibility, spin-state equilibria, conformational heterogeneity, and retained axial water. Water-retaining or water-bridged complexes can also produce type II-like spectra [4,5,7,8].

The term *heme chelation* should generally be avoided in this context. Most azoles, pyridines, and isonitriles act as monodentate axial ligands rather than chelators, because they bind iron through one donor atom. Nitriles are best regarded as context-dependent pharmacophoric, electronic, and orientational motifs rather than canonical direct axial ligands. Accordingly, *heme-binding group*, *heme-iron-coordinating group*, and *axial ligand* are more precise terms.

This focused mini-review addresses the following question: Which chemical groups genuinely function as heme-iron-interacting motifs in small-molecule CYP inhibitors, what evidence is required to distinguish direct axial coordination from water-mediated heme perturbation or metabolism-dependent inhibition, and how can these distinctions guide the design of selective CYP19A1 ligands? Selectivity is a central objective rather than a secondary optimisation parameter. The same distal heme-iron site is accessible in numerous CYP enzymes, so a donor group that improves CYP19A1 affinity may also inhibit drug-metabolising or other steroidogenic CYPs. Such off-target activity can compromise the interpretation of aromatase-directed effects and create drug–drug interaction or endocrine liabilities. The design problem is therefore to combine a productive heme-directed interaction with recognition features that favour the CYP19A1 binding cavity over those of other CYP isoforms [3,9,10,11].

To address this question, we critically compare established and putative heme-binding groups, discuss the interpretative limits of ligand-induced spectral responses, and outline an evidence framework integrating enzymology, spectroscopy, structural biology, and QM/MM calculations. CYP19A1 is considered as a mechanistically informative medicinal-chemistry case study rather than as a comprehensive review of aromatase inhibitor discovery. The discussion is deliberately restricted to small-molecule CYP inhibitors and does not attempt to review heme proteins more broadly.

## 2. Heme Coordination in CYP Enzymes

### 2.1. Electronic and Structural Basis

Cytochrome P450 enzymes differ from globins because their proximal heme ligand is a conserved cysteinate thiolate rather than a neutral histidine. The strong σ- and π-donor properties of this thiolate generate the *push effect*, which modulates the Fe(III)/Fe(II) redox equilibrium and promotes heterolytic O–O bond cleavage in the hydroperoxo-ferric intermediate, leading to Compound I formation. This electronic contribution is central to oxygen activation and CYP catalytic versatility [1,2,12]. 

**Figure 1:**
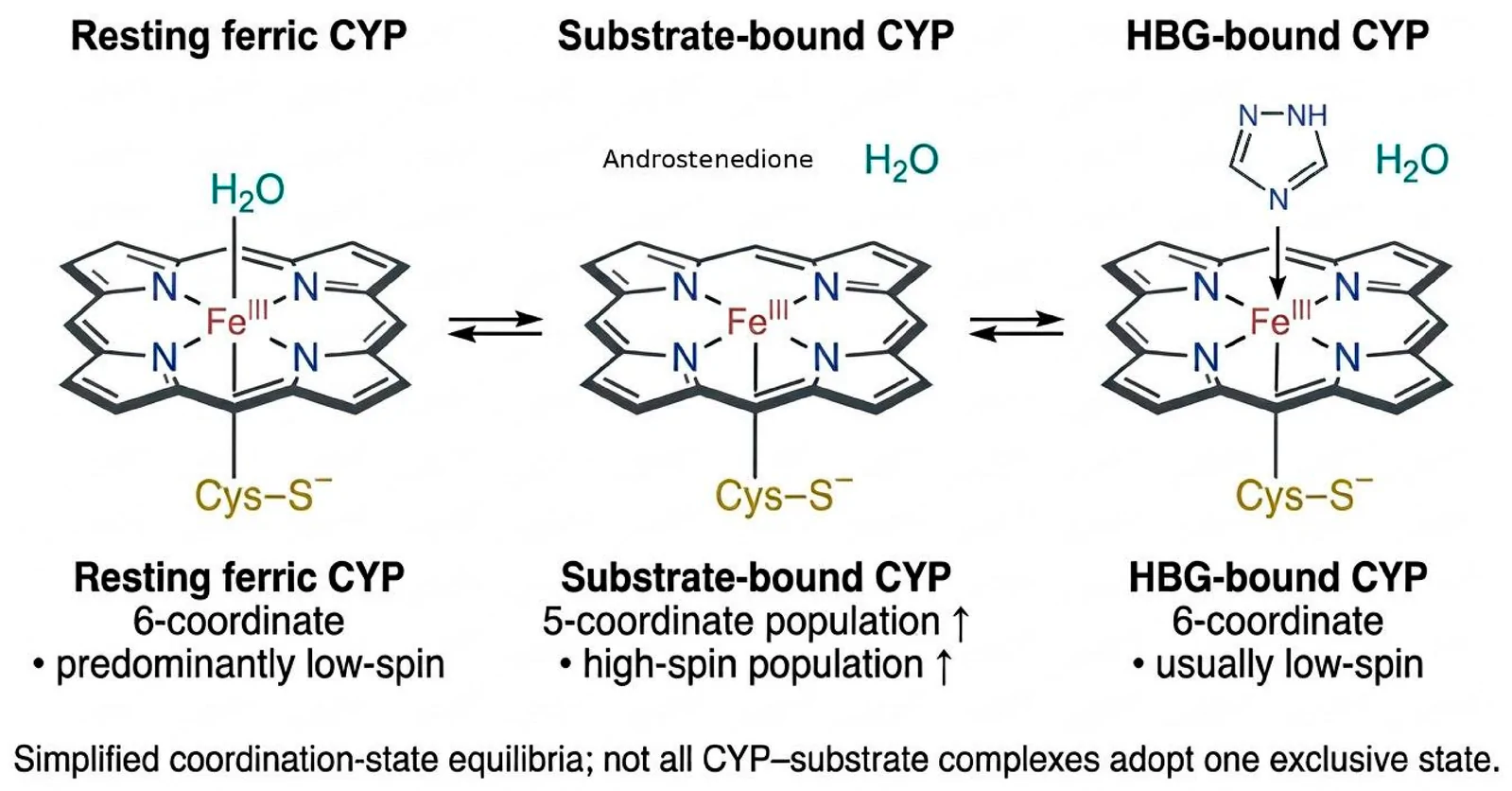
Simplified changes in distal heme coordination upon binding of a substrate or a heme-binding group (HBG) to a cytochrome P450 enzyme. In the resting ferric enzyme, the heme iron is commonly coordinated by four porphyrin nitrogens, the proximal cysteinate thiolate and distal water, giving a predominantly six-coordinate, low-spin state. Binding of many substrates perturbs or displaces distal water and increases the population of five-coordinate, high-spin heme without the substrate directly coordinating iron. An appropriately oriented nitrogen-containing HBG can instead replace distal water and form a six-coordinate Fe(III)–N complex, usually associated with a low-spin state. The panels depict simplified coordination-state equilibria; not all CYP–substrate complexes adopt one exclusive state [2,3,5].

In substrate-free ferric CYPs, water commonly occupies the distal sixth coordination site, producing a predominantly six-coordinate, low-spin state. A direct heme-binding group (HBG) must compete with this aqua ligand and access the distal heme face. Productive axial ligation requires suitable Fe–donor distance and approximately axial geometry, but the final arrangement is shaped by the protein environment, heme distortion, ligand flexibility, and possible water-mediated interactions [3,4,13]. Donor strength or basicity alone does not predict heme coordination. Even a strong Lewis base may fail to bind iron if its scaffold does not position it appropriately. The recognition scaffold controls donor presentation, water displacement or perturbation, and non-covalent interactions within the substrate-binding cavity and access channel. The substrate-induced change in distal coordination and the contrasting inhibitor-bound states are illustrated in Figure 1. Here, the donor vector denotes the three-dimensional direction in which an accessible donor-atom lone pair is presented from the bound molecular scaffold towards the distal heme-iron site. It depends not only on the identity of the donor atom, but also on its position in the ring, the attachment point, linker geometry and the ligand’s bound conformation. Two analogues bearing the same heterocycle may therefore differ substantially in their ability to approach iron along a productive axial trajectory [3,4,9].

Consequently, the same HBG may show different affinity, spin-state effects, spectral behaviour, and inhibitory potency across CYP isoforms because of variations in active-site volume, channel architecture, electrostatics, heme environment, and conformational dynamics [3,4,9,14,15,16].

### 2.2. Type I and Type II Spectral Responses

A type I spectral response is generally associated with a non-coordinating ligand that displaces or destabilises distal water, increasing the five-coordinate, high-spin ferric population. It typically displays a positive band near 390 nm and a negative band near 420 nm. Type I binding may be compatible with substrate-like positioning but does not establish productive turnover or a unique binding pose [17]. A type II response is usually associated with stabilization of a six-coordinate, low-spin ferric complex, often through direct coordination of a nitrogen donor. Imidazoles, triazoles, pyridines, and selected amines commonly produce such spectra, with a red-shifted positive band at approximately 425–440 nm [6]. Such complexes can inhibit catalysis by perturbing the heme environment and the catalytic cycle; however, their reduction behaviour is ligand- and isoform-dependent [7,17]. However, type I and type II spectra are phenomenological rather than definitive structural assignments. Mixed spectra may reflect partial spin-state conversion, multiple poses, incomplete coordination, or retained heme-bound water [4]. Therefore, UV–Vis spectroscopy should be complemented by EPR/HYSCORE/ENDOR, resonance Raman spectroscopy, structural biology, or benchmarked QM/MM calculations [3,4,6,8,13]. Importantly, these mechanisms may produce overlapping spectral phenotypes; therefore, neither the presence of a putative donor atom nor a type II difference spectrum alone establishes a defined direct Fe–donor coordination mode.

## 3. Direct, Reversible Heme-Binding Groups

### 3.1. Imidazole and Benzimidazole

Imidazole is a classical nitrogen donor and one of the most extensively validated heme-binding groups (HBGs) in CYP inhibition. Its pyridine-like nitrogen can coordinate ferric heme iron, stabilising a six-coordinate, low-spin complex that commonly produces a type II spectral response. Imidazole-containing antifungals, including ketoconazole, miconazole, clotrimazole, and econazole, demonstrate how an azole donor combined with a hydrophobic recognition scaffold can yield high-affinity CYP binding. In CYP3A4, such compounds commonly bind through direct heme coordination and may cause clinically relevant inhibition of drug metabolism [18,19]. In some chemotypes, reversible azole-mediated type II binding may coexist with metabolism-dependent heme interactions, illustrating that the spectral classification of the parent ligand does not by itself define the complete inhibitory mechanism [20].

A major limitation of imidazole donors is CYP promiscuity, particularly against CYP3A4, with consequent drug–drug interaction liability. Benzimidazoles provide larger, more hydrophobic scaffolds with distinct electronic properties and pKa values. For both classes, protonation state, tautomerism, substituent pattern, and donor accessibility should be considered. A docking pose is chemically meaningful only if the relevant bound microstate contains an unprotonated donor capable of approaching the heme iron along a feasible axial trajectory.

### 3.2. 1,2,4-Triazoles

1,2,4-Triazole is the best established HBG among nonsteroidal aromatase inhibitors and occurs in letrozole, anastrozole, vorozole, and numerous antifungal CYP inhibitors. Compared with imidazole, its additional ring nitrogens alter electronic distribution, basicity, hydrogen-bonding properties, and solvation behaviour, enabling modulation of affinity and CYP isoform selectivity [21]. The relevance of 1,2,4-triazoles to CYP19A1 is illustrated by the clinically used nonsteroidal aromatase inhibitors anastrozole and letrozole. In these compounds, the triazole ring is regarded as the principal heme-interacting motif, whereas the remaining substituents primarily govern recognition within the hydrophobic steroid-binding cavity, ligand orientation, and selectivity. The available evidence for the identity of the coordinating triazole nitrogen differs between compounds and is considered in detail in Section 5.2 [22,23].

### 3.3. 1,2,3-Triazoles

1,2,3-Triazoles are attractive modular motifs for analogue generation. They have been less represented in CYP inhibitor design than imidazoles and 1,2,4-triazoles. Their lower heme affinity has been attributed partly to an unfavourable entropic contribution associated with solvation, rather than solely to weaker intrinsic Fe–N bonding [8]. They should not automatically be described as direct axial Fe ligands. In a CYP3A4 study of a specific click-derived analogue, continuous-wave EPR and HYSCORE data indicated that the triazole did not displace the sixth axial water ligand. Instead, it formed a low-spin, water-bridged complex with heme-bound water [8]. Thus, 1,2,3-triazoles may form water-retaining complexes in a scaffold- and CYP-dependent manner; this observation should not be generalised to all members of the class or assumed to predict binding in CYP19A1 [8]. Accordingly, 1,2,3-triazoles are best viewed as tunable heme-environment-interacting motifs rather than universally direct HBGs. Their behaviour depends on substitution pattern, linker geometry, local hydration, and CYP conformational properties. Regioisomerism and donor presentation should therefore be assessed experimentally using spectral titrations, EPR-based methods, or structural studies.

### 3.4. Pyridine and Diazines

Pyridine is a compact, chemically stable N(sp^2^) donor and a well-established component of CYP inhibitors. Metyrapone is a classic example: CYP3A4 structures show direct coordination of a pyridyl nitrogen to heme iron, with an Fe–N distance of approximately 2.3 Å. Molecular-dynamics simulations proposed a stabilising interaction between the metyrapone carbonyl group and Ser119, but subsequent high-resolution crystallography did not identify a stable direct or water-mediated Ser119–metyrapone contact [24,25,26]. Pyridine-containing frameworks can also potently inhibit CYP19A1. Pyridine-based dual-binding-site aromatase inhibitors have achieved subnanomolar activity, supporting pyridine as a viable alternative to an azole in this inhibitor series, although the proposed Fe–N geometry is based primarily on modelling rather than atom-specific structural evidence [27]. Pyridazine, pyrimidine, and pyrazine offer further opportunities to tune donor basicity, polarity, and donor-site identity. Their design should focus on regioisomerism, protonation state, donor accessibility, and binding geometry. The relevant question is whether an unprotonated ring nitrogen can access the distal heme site in the dominant bound microstate.

### 3.5. Anilines and Related Aromatic Amines

Aniline-containing compounds have been used as simple nitrogen-donor ligands and can inhibit selected CYP enzymes. For example, 4-cyclohexylaniline inhibits aromatase in human placental microsomes [14,28]. However, aromatic amines are not intrinsically optimal drug-like HBGs. Their donor properties depend on substitution, protonation state, and the active-site environment, and some aromatic amines may undergo CYP-mediated N-hydroxylation to arylhydroxylamines, which can yield *nitroso* species capable of forming heme-associated complexes [29,30,31,32]. Aniline-containing ligands should therefore be evaluated for reversible inhibition and time-, concentration-, and NADPH-dependent inactivation. A nitroso intermediate or other reactive metabolite should be considered a compound-specific possibility requiring experimental confirmation, rather than an inevitable outcome for all aromatic amines.

### 3.6. Thiazoles and 1,2,3-Thiadiazoles

Sulfur- and nitrogen-containing five-membered heterocycles form a relatively unpredictable class of potential HBGs. Their electronic properties, donor atom, and heme interaction depend on substitution, oxidation state, protonation, and CYP context. A seminal study of 1,2,3-thiadiazoles reported isoform-dependent type I and type II responses; a fused bicyclic analogue also caused mechanism-based inactivation of CYP2E1 and CYP2B4. Inhibitory potency was not simply correlated with spectral dissociation constants or spectral classification [33]. The presence of several heteroatoms does not itself establish a specific Fe–N or Fe–S coordination mode. Thiazoles and thiadiazoles should therefore be described as putative or context-dependent HBGs unless supported by spectroscopy, structural biology, or carefully benchmarked QM/MM calculations.

### 3.7. Hydroxamates and Oxygen Donors

Hydroxamates are established bidentate metal-binding groups in zinc-dependent enzymes, including histone deacetylases and matrix metalloproteases. In CYP systems, they should not be considered canonical distal axial ligands equivalent to imidazoles, triazoles, or pyridines. Hydroxamate-containing compounds may inhibit CYPs, particularly CYP3A4, and adopt conformations compatible with heme-proximal interactions; however, inhibition or spectral perturbation does not establish direct axial O→Fe coordination. Quantum-mechanical and structural analyses indicate that these interactions are conformation-dependent and that an *s-trans* hydroxamate conformation is favoured in the studied complexes [34]. Hydroxamates are relevant because they may cause unwanted CYP inhibition in compounds developed for non-heme metalloproteins and broaden the chemical space of iron-interacting motifs. Their purposeful use in CYP19A1 design would require extensive validation because polarity and desolvation cost may limit productive binding in the hydrophobic aromatase active site.

### 3.8. Nitriles: Pharmacophoric Groups Rather than Default Fe Ligands

Nitriles require cautious interpretation in CYP inhibitor design. The nitrile nitrogen is weakly basic, and direct nitrile coordination to CYP heme iron is not broadly established as a dominant binding mode of clinically used CYP inhibitors. Nitriles are small, linear, and strongly dipolar groups that can influence molecular shape, electronic distribution, dipole-driven recognition, and—in favourable geometries—hydrogen-bond acceptance. Their effects on lipophilicity and affinity remain scaffold-dependent [35,36]. The absence of a general nitrile-to-heme binding rule should not be interpreted as chemical impossibility. In a synthetic iron(II) tetraphenylporphyrin complex, benzonitrile ligates iron through its nitrile nitrogen, demonstrating that an appropriately accessible metal centre and ligand geometry can support direct nitrile coordination [37]. This model-system observation cannot, however, be transferred directly to the thiolate-ligated, predominantly ferric CYP active site, where a nitrile-bearing inhibitor must also adopt a suitable pose and compete with distal water. For anastrozole, atom-specific HYSCORE evidence instead assigns the heme-coordinating atom to N4 of its 1,2,4-triazole ring [22]. Thus, its cyano substituents should not be labelled axial ligands without independent, compound-specific evidence. Nitrile coordination is possible in selected coordination environments, but it is not a default assignment for nitrile-containing CYP inhibitors.

This distinction is particularly relevant to nonsteroidal aromatase inhibitors. Letrozole and anastrozole both contain nitrile groups in addition to a 1,2,4-triazole moiety [21,22,38]. In this setting, nitriles should primarily be regarded as elements that influence molecular shape, dipole distribution, hydrophobic complementarity, and ligand orientation rather than as default axial heme ligands. The assignment of the coordinating motif in these inhibitors is discussed in Section 5.2.

### 3.9. Isonitriles

Isonitriles, represented as R–N≡C, differ from nitriles because they coordinate metal centres through carbon. Alkyl isonitriles can ligate CYP heme iron in both ferric and ferrous states. Steroid-derived isonitriles provide proof of principle that a strong HBG can be incorporated into a steroid-like scaffold while retaining selectivity for particular CYP enzymes. Reported studies examined steroidogenic CYP17A1 and bacterial CYP106A2, alongside CYP3A4 and CYP2D6 [39]. These findings indicate that heme coordination strength alone does not determine CYP promiscuity; selectivity also depends on scaffold recognition, active-site architecture, and access-channel complementarity. Available evidence concerns CYP17A1, CYP106A2, CYP3A4, and CYP2D6, and indicates that productive ligation depends strongly on the position and stereochemistry of the isonitrile group. Isonitrile-containing compounds should therefore be assessed carefully for chemical reactivity, metabolic stability, broad CYP inhibition, cellular selectivity, and safety. Taken together, the chemical classes discussed in this section differ substantially in their propensity for direct heme-iron ligation, the strength of evidence supporting a defined coordination mode, and their medicinal-chemistry liabilities. Their principal features and the experimental approaches required for mechanistic validation are summarised in Table 1.

**Figure 2:**
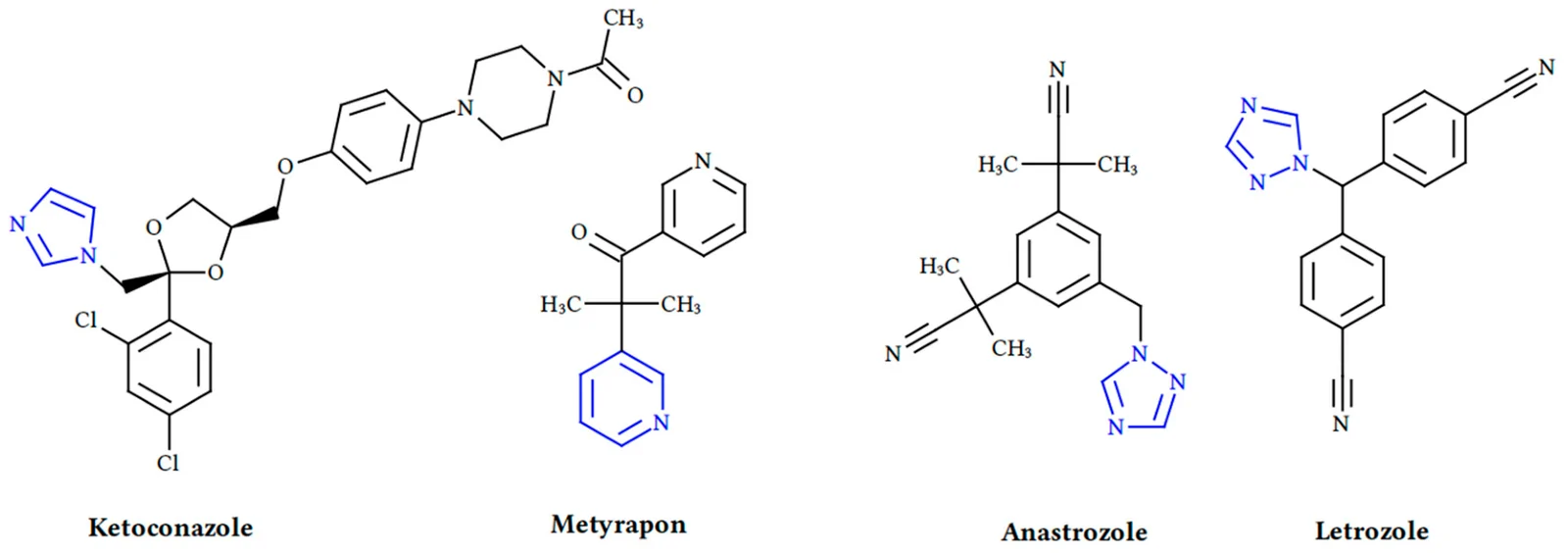
Examples of drug formulas with marked fragments involved in interactions with heme iron.

This comparison also emphasizes the need to distinguish reversible parent–ligand interactions with the heme environment from metabolism-dependent inhibition, including mechanism-based inactivation and metabolic-intermediate complex formation. Representative heme-interacting motifs in small-molecule CYP inhibitors are shown in Figure 2.

## 4. Mechanism-Based Inactivation Is Distinct from Direct Reversible Heme Ligation

Direct, reversible heme ligation should be distinguished from mechanism-based inactivation (MBI). In classical CYP-mediated MBI, the parent compound is metabolised by the target enzyme to form a reactive intermediate. This species can reduce catalytic activity through covalent modification of the apoprotein, covalent modification or destruction of heme, or formation of a metabolic-intermediate complex (MIC) with heme iron. Covalent protein or heme damage is generally irreversible under conventional in vitro recovery conditions, whereas an MIC is more appropriately described as quasi-irreversible because it may dissociate or be chemically reversed slowly under suitable conditions [29,40,41,42]. The electronic nature of coordination may also differ between reversible inhibitor complexes and quasi-irreversible metabolic-intermediate complexes, particularly after reduction of the heme iron [43,44].

The spectral and temporal behaviour provides a useful first distinction. A direct, reversible parent–ligand complex may generate a type II difference spectrum immediately upon ligand addition, typically with a positive feature in the approximately 425–440 nm region; this response alone does not establish a direct Fe–N bond [4,6]. An MIC instead requires CYP-dependent generation of a coordinating metabolite and is assessed through its development during NADPH-supported turnover, loss of activity over time and persistence after removal or dilution of the parent compound [29,40]. For selected methylenedioxyphenyl-derived carbene MICs, a feature near 455 nm together with a band or shoulder near 425–427 nm has been reported [45]. Peak positions and intensities depend on the system and intermediate: neither a 455 nm feature nor a type II spectrum should substitute for NADPH controls, recovery experiments and, where feasible, metabolite or adduct identification.

MBI is commonly associated with time-, concentration-, and NADPH-dependent loss of enzyme activity. These features alone, however, do not establish the molecular mechanism or the site of damage. A robust assessment should include preincubation studies with and without NADPH, determination of *K_I_* and *k_inact_*, dilution or dialysis recovery experiments, and, where feasible, spectroscopic monitoring and LC–MS-based detection of heme or apoprotein adducts [46].

### 4.1. Nitroso Intermediates Formed from Amines

Selected aliphatic and aromatic amines may undergo CYP-mediated oxidation through hydroxylamine and nitroso intermediates. For aliphatic amines, nitrosoalkane metabolites can coordinate heme iron and form metabolic-intermediate complexes; aromatic amines may analogously generate nitrosoarene-type species. The efficiency, reversibility, and toxicological consequences of these pathways depend on the parent structure, CYP isoform, redox conditions, and active-site environment. A single endpoint IC_50_ value cannot distinguish reversible inhibition, substrate turnover, or inhibition mediated by a reactive nitroso metabolite. Time-dependent inhibition experiments, NADPH controls, recovery studies, and direct metabolite or MIC evidence are therefore required [30,31].

### 4.2. Carbene–Heme Complexes from Methylenedioxyphenyl Groups

**Figure 3:**
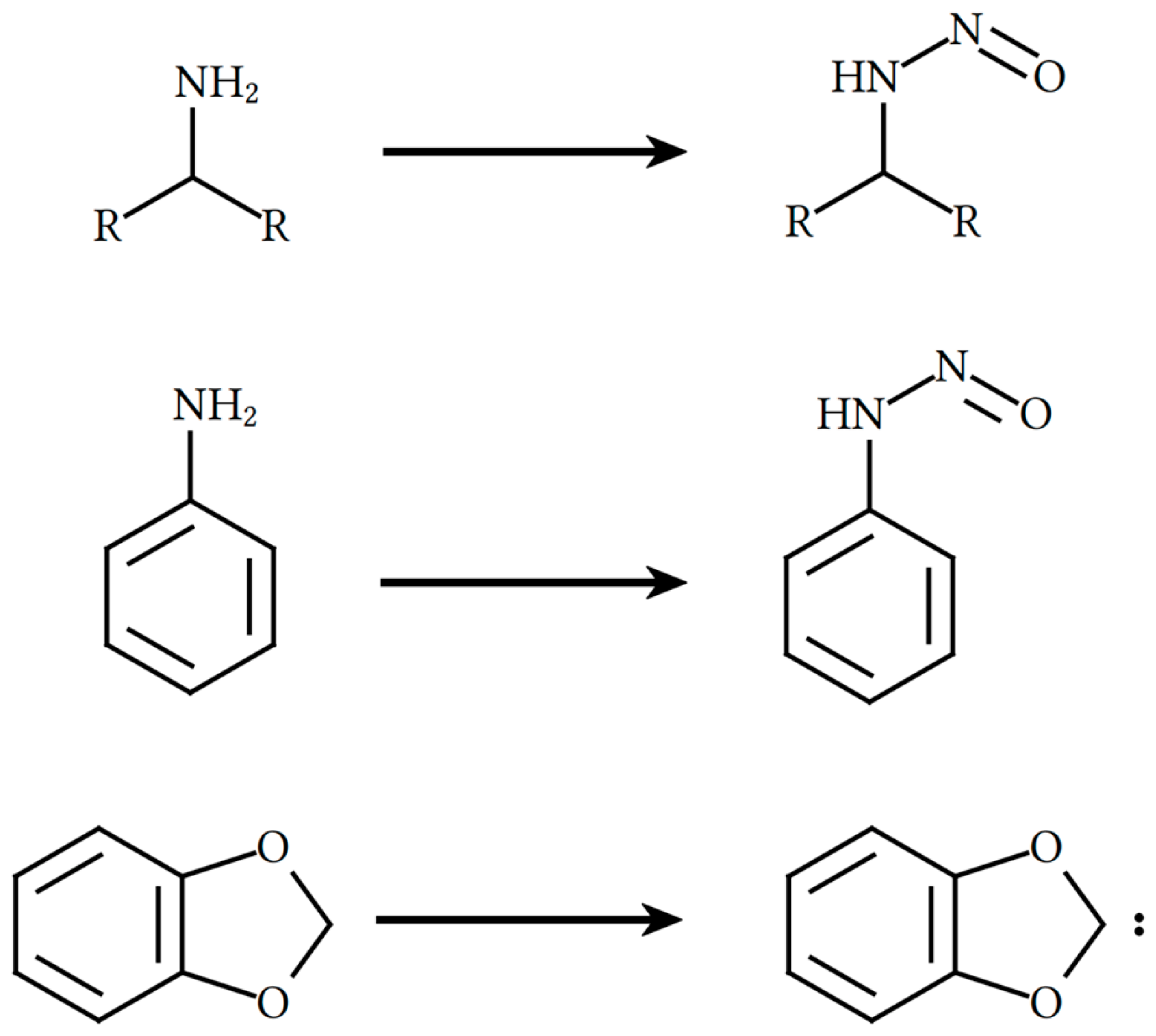
Representative metabolic transformations of amine- and methylenedioxyphenyl-containing compounds. CYP-mediated oxidation of selected amines can generate hydroxylamine and nitroso derivatives, whereas metabolism of methylenedioxyphenyl-containing compounds can generate carbene intermediates. The schemes illustrate possible pathways of intermediate formation; they do not depict heme binding or establish MIC formation. The pathways are compound-dependent [29,30,31,45,47,48].

Methylenedioxyphenyl (MDP) groups can generate CYP-mediated carbene intermediates that coordinate heme iron and form carbene–heme MICs. The initially formed carbene–heme complex has been proposed to involve an Fe(III)–carbene species and to evolve into a more slowly reversible Fe(II)–carbene complex associated with quasi-irreversible inhibition. MDP-containing compounds may therefore show reversible inhibition by the parent molecule and time-dependent inhibition after metabolic activation. Their evaluation should include NADPH-dependent preincubation, recovery after dilution or dialysis, MIC spectral monitoring, and, where possible, direct metabolite or adduct identification [47,48]. Direct HBGs act as parent-molecule ligands, whereas MBI precursors exploit CYP turnover to generate a reactive or strongly coordinating species. Although therapeutically useful in selected settings, MBI carries a greater risk of persistent off-target CYP inhibition and drug–drug interactions. Representative CYP-dependent transformations of selected amine- and methylenedioxyphenyl-containing compounds are shown in Figure 3.

## 5. CYP19A1 as a Model Target

### 5.1. Why Aromatase Is an Instructive Model

Aromatase (CYP19A1) catalyses the terminal conversion of androgens into estrogens and is a clinically validated target in estrogen receptor-positive breast cancer. Its compact, predominantly hydrophobic active site accommodates the steroid nucleus of androstenedione above the heme, while residues in the I-helix and access-channel regions contribute to substrate recognition and catalytic positioning. This combination of a steroid-recognition pocket and a heme-proximal coordination site makes CYP19A1 a useful model for investigating HBG identity, ligand geometry, and scaffold-dependent selectivity [23,49,50].

Clinically used aromatase inhibitors illustrate two mechanistically distinct approaches to CYP19A1 inhibition. Exemestane is a steroidal substrate analogue that undergoes CYP19A1-dependent activation and produces time-dependent, functionally irreversible inhibition. In contrast, the nonsteroidal inhibitors anastrozole, letrozole, and vorozole are generally treated as reversible competitive inhibitors whose azole-containing pharmacophores perturb the heme environment. This contrast makes CYP19A1 a useful model for distinguishing parent–ligand heme interactions from metabolism-dependent inactivation [21,51,52,53,54]. Their initial ferric-heme spectra also differ. Exemestane produces a substrate-like type I response in human aromatase, including a reported Soret shift from approximately 418 to 394 nm, consistent with an increased high-spin population following perturbation or displacement of distal water. Anastrozole instead produces a low-spin, type II response, with a reported Soret shift from approximately 418 to 422 nm; HYSCORE experiments assign coordination to triazole N4 under the examined conditions [22,55]. These observations describe the initial ligand-bound states. Exemestane’s subsequent metabolism-dependent, functionally irreversible inhibition must be established from time-dependent functional and mechanistic evidence, not inferred from its type I spectrum alone [53,55].

### 5.2. Correct Assignment of the Coordinating Motif

The nitrile groups of anastrozole and letrozole should not be assigned as axial heme ligands solely from their presence in the molecular structures [22,38]. In both compounds, the 1,2,4-triazole moiety is the most plausible and mechanistically relevant iron-interacting pharmacophore, whereas nitrile-bearing substituents are more likely to contribute to ligand orientation, electronic distribution, hydrophobic complementarity, and interactions with the surrounding protein environment [21,22,38].

For anastrozole, HYSCORE spectroscopy provides direct evidence that N4 of the 1,2,4-triazole ring coordinates the aromatase heme iron under the examined conditions. Structural models have additionally proposed interactions between triazole N1 and Thr310 and between a cyano group and Asp309. These secondary contacts should be interpreted as model-supported binding hypotheses rather than as direct structural observations [22,23]. Letrozole likewise contains a 1,2,4-triazole group that is widely regarded as its principal heme-interacting pharmacophore. Its two cyanophenyl substituents are expected to contribute predominantly to shape complementarity and ligand positioning in the aromatase active site. However, unlike anastrozole, the identity of the coordinating triazole nitrogen in letrozole has not been established by direct HYSCORE evidence in human aromatase. Its assignment is therefore based primarily on pharmacophore-based and mechanistic inference. The distinction is important: anastrozole provides stronger experimental support for assignment of the coordinating atom, whereas the corresponding structural interpretation for letrozole remains less direct [38].

### 5.3. Design Hypotheses for New CYP19A1 Ligands

A CYP19A1 design programme can be organised around matched molecular-pair series that retain a common aromatase-recognition scaffold while systematically varying donor identity, donor vector, and local electronic properties. Useful starting points include 1,2,4-triazoles, pyridines, selected diazines, imidazoles, and exploratory 1,2,3-triazole or isonitrile analogues. Such compounds should not be ranked solely according to the nominal donor strength of the heterocycle, because productive binding also depends on donor presentation, ligand microstate, active-site hydration, and recognition by the surrounding scaffold [3,4,8].

For CYP19A1, activity and selectivity are expected to depend on the combined effects of donor presentation, protonation and tautomeric state, ligand desolvation, interactions with the steroid-binding cavity, and complementarity with the access channel. The relevant design objective is therefore not simply to maximise heme affinity, but to identify scaffolds that present a suitable donor atom while avoiding broad inhibition of drug-metabolising and steroidogenic CYP enzymes [27]. As summarised in Table 1, 1,2,4-triazoles and pyridines represent the best established starting points for direct, reversible heme-iron coordination in CYP inhibitor design. Imidazoles may provide effective heme-binding interactions but often carry a greater risk of broad CYP inhibition, whereas 1,2,3-triazoles, sulfur-containing heterocycles, hydroxamates, nitriles and isonitriles require more context-dependent, compound-specific assessment of their coordination mode and selectivity liabilities.

A focused design matrix should evaluate:

- HBG identity, pKa, protonation state, and dominant tautomer;
- Linker length, rigidity, and donor vector;
- Substituents affecting donor basicity or steric accessibility;
- Nitrile replacements with hydrogen-bond acceptors, halogens, heteroaryls, or polar isosteres;
- 2-, 3-, and 4-pyridyl vectors where productive heme approach is feasible;
- Selectivity against drug-metabolising CYPs and steroidogenic CYPs, especially CYP17A1, CYP11B1, CYP11B2, CYP21A2, and CYP11A1 [10,11].

These design variables should be evaluated within an integrated workflow that links donor-group selection and scaffold optimization to functional inhibition kinetics, orthogonal assessment of heme interactions, early profiling of selectivity and time-dependent inhibition, and cellular target-engagement studies.

The goal should extend beyond lower IC_50_ values. More informative outcomes are structure–mechanism–selectivity relationships linking HBG class, spectral response, K*_i_*, time dependence, binding kinetics, supported Fe–donor geometry, and inhibition across drug-metabolising and steroidogenic CYP isoforms. Closely related steroidogenic CYP enzymes may also be intentionally co-targeted in selected therapeutic concepts; for example, dual CYP19A1/CYP11B2 inhibitors have been developed to combine aromatase inhibition with suppression of aldosterone synthase [56].

## 6. How to Validate an Fe–Donor Interaction

**Figure 4:**
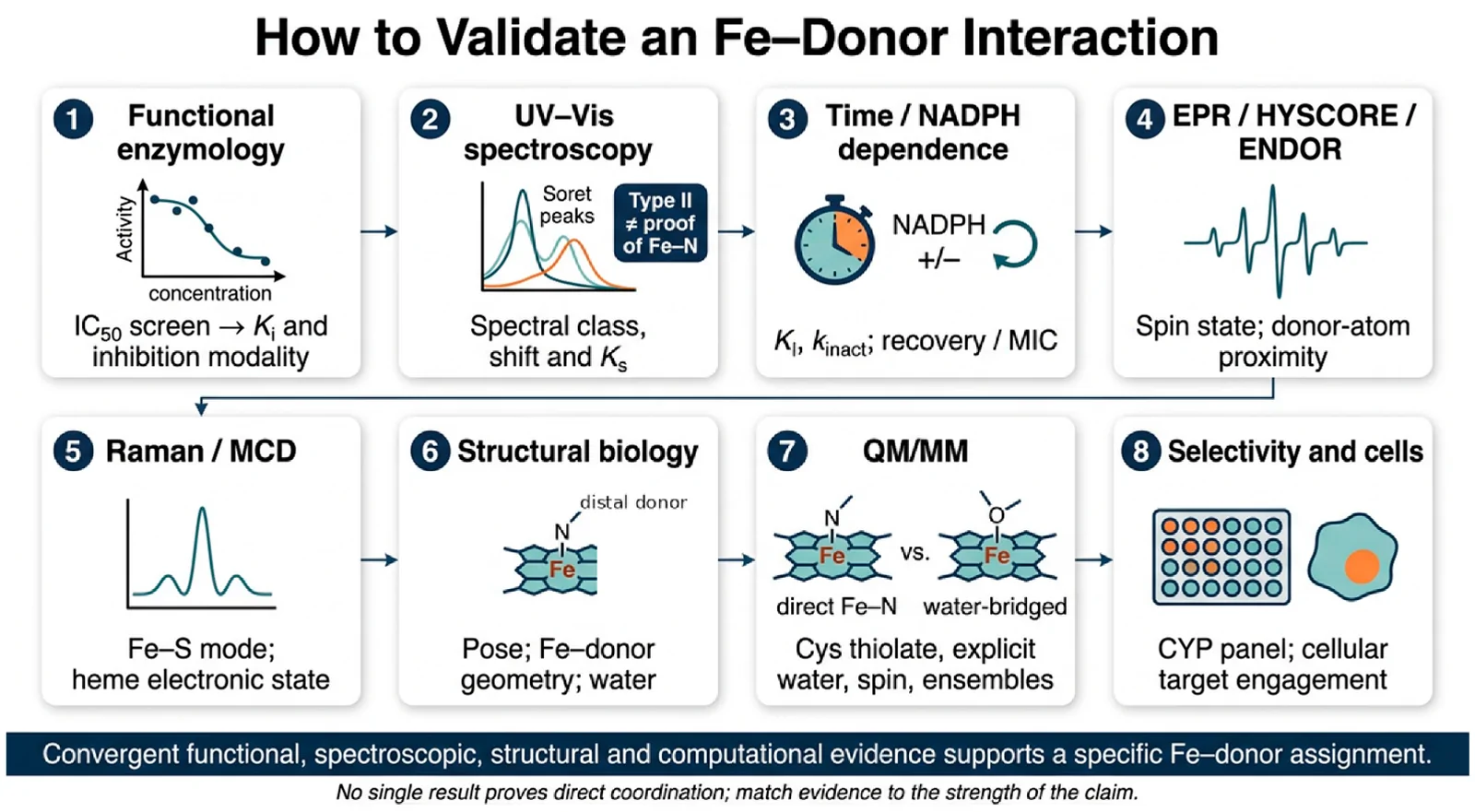
Complementary functional, spectroscopic, structural and computational approaches for assessing a proposed Fe–donor interaction. No single assay independently establishes a specific coordination geometry.

Assignment of a direct Fe–donor interaction requires a workflow in which each method addresses a distinct uncertainty. Functional assays establish inhibitory potency and modality, spectroscopic methods report ligand-induced changes in the heme environment, structural approaches define plausible ligand geometries, and QM/MM calculations test whether proposed coordination modes are chemically consistent in the protein environment. No individual method is sufficient in every system; the required level of validation should reflect the strength of the mechanistic claim (Figure 4).

### 6.1. Functional Enzymology

IC_50_ measurements are useful for initial potency ranking but should not be interpreted as direct measures of affinity or heme coordination. IC_50_ values depend on substrate and enzyme concentrations, incubation time, assay format, and preincubation conditions. Hits from IC_50_ screens should therefore be characterized using an inhibition model appropriate to the experimental system. For reversible inhibitors, *K_i_* should be determined from substrate–velocity measurements across suitable substrate and inhibitor concentrations. The reversible inhibition modality—competitive, non-competitive, uncompetitive, or mixed—should be established using appropriate kinetic models. Time-dependent inhibition should be assessed separately through matched preincubation experiments with and without NADPH, followed by estimation of *K_I_* and *k_inact_* when indicated.

### 6.2. UV–Vis Difference Spectroscopy

UV–Vis difference spectroscopy provides a rapid method for assessing ligand-induced perturbation of the CYP heme environment. Full ligand titrations should determine the apparent spectral dissociation constant, *K_s_*, Soret-shift magnitude and wavelength, and spectral class: type I, type II, mixed, reverse type I, or atypical. Imidazole, 1,2,4-triazole, pyridine, and aniline can serve as reference ligands for a given CYP isoform [3]. Type II-like spectral changes should be reported as evidence for stabilisation of a low-spin heme environment and interpreted together with the full binding isotherm, the apparent spectral dissociation constant, and the magnitude of the induced Soret shift. When direct coordination is proposed, the spectral result should be treated as a hypothesis-generating observation that defines the need for orthogonal validation, rather than as a stand-alone structural assignment.

### 6.3. Time Dependence and NADPH Dependence

Potential mechanism-based inactivation should be evaluated by preincubating the inhibitor with the relevant CYP system in the presence and absence of NADPH or an NADPH-regenerating system. An initial screen may compare direct inhibition with inhibition after matched preincubations. A lower IC_50_ after NADPH-supported preincubation indicates time-dependent inhibition liability. However, an IC_50_ shift alone does not establish mechanism-based inactivation. Follow-up experiments should determine time and concentration dependence of inactivation, allowing estimation of *K_I_* and *k_inact_*. Dilution or dialysis recovery experiments help distinguish persistent inactivation from reversible binding by residual parent compound or metabolite. Where appropriate, detection of metabolic-intermediate complexes and LC–MS analysis of heme or apoprotein adducts should define the underlying mechanism [40,57,58].

### 6.4. EPR, HYSCORE, and ENDOR

EPR-based methods provide information on CYP heme spin state, coordination environment, and electronic structure. HYSCORE and ENDOR can reveal hyperfine interactions between paramagnetic heme iron and nearby ligand nuclei, providing strong evidence for the identity and proximity of a proposed donor atom. HYSCORE analysis was instrumental in identifying N4 of anastrozole’s 1,2,4-triazole ring as the iron-coordinating atom in human aromatase [3,22]. These methods should be interpreted alongside functional and structural evidence. Conclusions depend on experimental conditions, spin-state populations, isotope labelling where available, and robust spectral simulation. Nevertheless, they can help distinguish direct coordination from some water-mediated arrangements.

### 6.5. Resonance Raman and Magnetic Circular Dichroism

Resonance Raman spectroscopy can resolve marker bands associated with porphyrin core size, spin state, and coordination state. It can also probe the ν(Fe–S) stretching mode, which reports on coupling between heme iron and proximal cysteinate thiolate. Ligand-induced changes may support altered axial ligation, water displacement, or modification of the heme electronic environment. Magnetic circular dichroism is useful for analysing low- and high-spin populations, charge-transfer transitions, and heme electronic structure. Together, resonance Raman and MCD complement UV–Vis and EPR measurements by helping distinguish ligand-induced spin-state changes from direct changes in axial coordination [3,13].

### 6.6. Structural Biology

Well-resolved X-ray crystallography or cryo-EM can provide spatial evidence for ligand orientation, water retention or displacement, and plausible Fe–X distances. Such conclusions are most persuasive when ligand occupancy is high and density supports a single pose. Structural evidence nevertheless requires caution. In crystallography, radiation-induced heme reduction, partial ligand occupancy, conformational heterogeneity, and limited resolution can complicate coordination-state assignment [59]. In cryo-EM, local active-site resolution, compositional heterogeneity, and flexible ligand density may limit confidence in precise Fe–X distances. Structural observations should therefore be integrated with solution-state spectroscopy and enzyme kinetics.

### 6.7. QM/MM Calculations

QM/MM calculations can test whether a proposed pose is chemically and energetically consistent with direct heme coordination. Robust protocols should consider ligand protonation and tautomeric states, explicit heme-bound water, alternative poses, protein conformational sampling, and relevant iron spin states [60]. Relative free energies are preferable to isolated gas-phase interaction energies. A single gas-phase HBG binding-energy calculation is unlikely to rank ligands reliably in a protein environment because it neglects water displacement, electrostatics, desolvation, conformational entropy, access-channel effects, and recognition-scaffold contributions [61]. As a practical minimum, the QM region should represent the heme iron, porphyrin, proximal cysteinate thiolate and proposed distal donor in a manner that preserves the thiolate’s electronic contribution to the heme (“push effect”) [2,12,60]. The distal-water-bound reference state and relevant nearby waters should be treated explicitly when testing water displacement versus water-retaining binding. Directly coordinated, water-bridged and non-coordinated poses should then be compared across plausible ligand protonation states, iron spin states and more than one protein/ligand conformation [5,8,60,61]. An Fe–donor distance or interaction energy obtained from one optimised, water-free pose is not a binding free energy and may give a misleading coordination preference; the comparison should include water displacement, protein relaxation and, where feasible, conformational sampling. QM/MM is therefore most informative when constrained or benchmarked by spectroscopic and structural observations [60].

### 6.8. Selectivity, Cellular Activity, and Target Engagement

Biochemical CYP19A1 activity should be accompanied by broad CYP profiling. Drug–drug interaction (DDI) assessment should include CYP1A2, CYP2B6, CYP2C8, CYP2C9, CYP2C19, CYP2D6, and CYP3A for reversible and, where appropriate, time-dependent inhibition. Profiling against CYP17A1, CYP11B1, CYP11B2, CYP21A2, and CYP11A1 is also important [10,11]. Cellular assays should confirm that biochemical inhibition translates into reduced aromatase activity in CYP19A1-expressing systems. Preferred readouts include LC–MS/MS measurement of androgen-to-estrogen conversion or validated estrogen-response assays. Cellular potency should be interpreted alongside intracellular exposure, CYP19A1 expression, cytotoxicity, membrane effects, and inhibition of other steroidogenic enzymes. Weak cellular estrogen suppression despite strong purified-enzyme activity may indicate poor permeability, nonspecific binding, rapid metabolism, or an incorrect binding hypothesis.

## 7. Conclusions

Heme-iron interactions are an important, but frequently overinterpreted, component of CYP inhibitor design. The available evidence supports imidazoles, 1,2,4-triazoles and pyridines as the most established starting points for direct, reversible axial coordination of the distal heme iron in CYP enzymes. By contrast, the behaviour of 1,2,3-triazoles, sulfur-containing heterocycles, hydroxamates, nitriles and isonitriles is more dependent on ligand structure, donor presentation, local hydration, CYP isoform and experimental conditions. These groups should therefore not be assigned a uniform coordination mode solely on the basis of heteroatom identity or inhibitory potency.

These established donors present distinct medicinal-chemistry trade-offs. Imidazoles are effective heme ligands, but an exposed imidazole nitrogen can favour inhibition of multiple CYP isoforms and consequently increase drug–drug interaction liability; protonation and tautomerism must also be considered [18,20]. A 1,2,4-triazole is a validated CYP19A1-directed motif, yet its use does not confer selectivity by itself: ring substitution, the accessible nitrogen and scaffold-controlled donor presentation remain decisive, and off-target CYP activity requires measurement [10,11,21,22]. Pyridines offer compact, synthetically versatile donors, but pyridyl substitution pattern and basicity can change the approach to heme, while direct Fe–N coordination may also occur in drug-metabolising CYPs [24,26,27]. Selection among these groups should therefore follow matched-series comparisons of potency, binding geometry, physicochemical behaviour and CYP-panel selectivity rather than a ranking based on donor strength alone.

A type II spectral response is useful evidence for stabilization of a low-spin heme environment, but it does not by itself establish a persistent direct Fe–donor bond. Direct reversible ligation must be distinguished from water-retaining or water-bridged heme interactions and from metabolism-dependent inhibition, including the formation of metabolic-intermediate complexes. The strength of the experimental evidence should match the precision of the mechanistic claim: inhibition kinetics and UV–Vis difference spectroscopy may support an operational classification of ligand binding, whereas assignment of a specific coordinating atom requires complementary evidence from EPR-based methods, resonance Raman spectroscopy, high-quality structural data, or spectroscopy-constrained QM/MM calculations.

CYP19A1 provides an instructive medicinal-chemistry case study because potent inhibition requires simultaneous optimisation of heme-proximal donor presentation, recognition of the hydrophobic steroid-binding cavity, ligand microstate, active-site hydration and selectivity against drug-metabolising and steroidogenic CYP enzymes. In this context, the nitrile substituents of anastrozole and letrozole should not be treated as default heme ligands; the 1,2,4-triazole motif is the more plausible heme-interacting pharmacophore, although atom-specific coordination assignments require direct experimental support.

Overall, the central question is not whether a compound contains a nominally basic heteroatom, but whether its dominant bound microstate can productively perturb or replace the distal heme-water environment in a specific CYP active site. For CYP19A1 ligand discovery, donor-group selection should therefore be guided not only by nominal Lewis basicity or a type II spectral response, but also by donor vector, scaffold recognition, active-site hydration, time-dependent inhibition liability and broad CYP selectivity. This integrated framework can help distinguish genuinely heme-directed, selective aromatase inhibitors from compounds whose apparent activity reflects nonspecific CYP binding or metabolism-dependent effects.

## 8. Future Prospects

An important unresolved question is how consistently donor-atom assignments made in solution translate into a single, structurally defined pose in CYP19A1. For anastrozole, HYSCORE supports coordination through triazole N4 [22], but an inhibitor-bound structure of sufficient quality to independently assess that atom’s position, the Fe–N geometry and distal-water occupancy would strengthen the combined mechanistic interpretation. Comparable atom-specific evidence remains limited for other proposed CYP19A1 ligands, particularly when alternative nitrogens, water-retaining poses or multiple microstates are possible [4,8,22]. Priority should therefore be given to matched analogue series examined by kinetics, UV–Vis and EPR-based spectroscopy, high-quality structural methods where feasible, and water-aware, ensemble-based QM/MM calculations. Such studies could test whether changes in the donor vector improve CYP19A1 selectivity without increasing inhibition of drug-metabolising or other steroidogenic CYP enzymes [10,11,60].

## Figures and Tables

**Table 1:** Heme-binding and heme-iron-interacting groups in small-molecule CYP inhibitors: coordination behaviour, medicinal-chemistry utility and validation priorities.

Chemical Class	Typical Donor Atom or Interaction	Evidence for Direct Heme Ligation	Medicinal-Chemistry Utility	Main Limitations or Liabilities	Priority Validation Approaches
Imidazoles and benzimidazoles	Pyridine-like N; usually monodentate axial N → Fe ligation	Strong for multiple CYP–ligand complexes; scaffold- and isoform-dependent	Compact and effective HBGs; broad synthetic accessibility; proven CYP affinity	Frequent broad CYP inhibition, notably CYP3A-mediated DDI liability; protonation and tautomerism affect donor availability	Enzyme kinetics, UV–Vis difference spectra, CYP selectivity panel; structural or EPR-based support where atom-specific assignment is claimed
1,2,4-Triazoles	Ring nitrogen; direct axial N → Fe ligation is common	Strong; clinically relevant examples include nonsteroidal aromatase inhibitors	Established HBGs; tunable electronics, polarity and donor vector; validated relevance to CYP19A1	Potential inhibition of drug-metabolising and steroidogenic CYPs; tautomeric state and coordinating N may be ambiguous	UV–Vis titration, *K_i_*, CYP profiling; HYSCORE/ENDOR or structural support for coordinating-N assignment
Pyridines and diazines	Ring N; often direct axial N → Fe ligation	Strong for pyridines; more context-dependent for diazines	Stable, synthetically versatile, easily varied electronic and geometric properties	Potential CYP promiscuity; basicity and regioisomerism strongly influence binding	Kinetics, spectral titration, broad CYP panel; structural studies or validated QM/MM for donor-vector analysis
1,2,3-Triazoles	Nitrogen-rich heteroarene; direct ligation or water-retaining/water-bridged arrangement	Context-dependent; direct Fe–N coordination should not be assumed	Highly modular and click-compatible; useful for rapid analogue generation	High desolvation cost in some contexts; uncertain donor identity; type II-like spectra may be misleading	Full spectral titration, EPR/HYSCORE/ENDOR, water-aware structural analysis or QM/MM
Anilines and related aromatic amines	Amino N; possible parent–ligand interaction; may yield nitroso metabolites	Variable and compound-specific	Simple, modifiable nitrogen-containing motifs; historically relevant to CYP19A1 inhibition	Possible CYP-mediated N-hydroxylation and nitroso-derived MIC formation; safety and TDI concerns	Reversible kinetics plus matched NADPH-dependent preincubation, *K_I_* and *k*_inact_ where appropriate, recovery and metabolite analysis
Thiazoles and 1,2,3-thiadiazoles	Possible N or S interaction; mode is structure- and CYP-dependent	Limited and context-dependent	Electronic diversity and access to underexplored HBG space	Donor identity uncertain; variable spectral phenotype; potential MBI for selected chemotypes	UV–Vis, NADPH/time-dependence testing, EPR or structural validation, metabolite characterization when indicated
Hydroxamates and related oxygen donors	O donor(s); heme-proximal interaction rather than canonical axial ligation	Limited for direct axial O→Fe ligation	Relevant for anticipating off-target CYP inhibition in metalloprotease/HDAC programmes	High polarity and desolvation cost; conformation-dependent interactions; direct O→Fe assignment often unsupported	CYP inhibition profiling, conformational analysis, structural data and QM/MM; avoid interpreting type II response alone as proof
Nitriles	Usually orientational, dipolar and hydrogen-bond-accepting motif rather than a canonical axial donor	Weak for direct CYP heme ligation	Small, metabolically robust and frequently useful for tuning shape, polarity and lipophilicity	Common risk of incorrectly assigning nitrile N as the heme ligand; effect strongly scaffold-dependent	Treat as a noncanonical HBG unless direct evidence is available; use structure/spectroscopy to establish donor identity
Isonitriles	Carbon donor; direct Fe–C coordination possible in ferric and ferrous CYP states	Strong in selected systems, but limited chemical space	Strong, unusual heme-binding motif; potential for scaffold-driven CYP selectivity	Chemical reactivity, metabolic stability, safety and broad CYP inhibition must be considered	Redox-state-sensitive spectroscopy, structural characterization, broad CYP/TDI profiling and cellular safety assessment

## Data Availability

No new data were created or analyzed in this study. Data sharing is not applicable to this article.
